# Ceragenins Prevent the Development of Murine Vaginal Infection Caused by *Gardnerella vaginalis*

**DOI:** 10.3390/ph17111445

**Published:** 2024-10-29

**Authors:** Urszula Wnorowska, Ewelina Piktel, Tamara Daniluk, Paulina Paprocka, Paul B. Savage, Bonita Durnaś, Robert Bucki

**Affiliations:** 1 Department of Medical Microbiology and Nanobiomedical Engineering, Medical University of Białystok, 15-222 Białystok, Poland; urszula.wnorowska@umb.edu.pl (U.W.); tamara.daniluk@umb.edu.pl (T.D.); 2 Independent Laboratory of Nanomedicine, Medical University of Białystok, 15-222 Białystok, Poland; ewelina.piktel@umb.edu.pl; 3 Department of Microbiology and Immunology, Institute of Medical Science, Collegium Medicum, Jan Kochanowski University in Kielce, 25-317 Kielce, Poland; paulina.paprocka@ujk.edu.pl (P.P.); bonita.durnas@ujk.edu.pl (B.D.); 4 Department of Chemistry and Biochemistry, Brigham Young University, Provo, UT 84602, USA; pbsavage@chem.byu.edu

**Keywords:** bacterial vaginosis, *Gardnerella vaginalis*, ceragenins, biofilm

## Abstract

**Background/Objectives:** Bacterial vaginosis (BV), an infection caused primarily by *Gardnerella vaginalis*, is the most prevalent vaginal infection. Although BV is often characterized by an asymptomatic course, it can lead to considerable health complications. Currently, BV therapy choices are limited, and available treatments are complicated by concerns about antibiotic resistance. Ceragenins, which together comprise an innovative class of low molecular-weight, cholic acid-based antibacterial agents, have emerged as potential alternatives to conventional treatments. **Methods:** This study investigates (i) the antibacterial activity of ceragenins against *G. vaginalis* in in vitro experimental settings at varied pH, and (ii) the effectiveness and anti-inflammatory properties of CSA-13 in a *G. vaginalis*-induced bacterial vaginosis animal model. **Results and Conclusions:** We demonstrate that ceragenins, particularly CSA-13, maintain their antibacterial efficacy throughout pH range of 4.5–7, with the highest activity observed at neutral pH (7.0). Additionally, in an animal model, beneficial effects of ceragenins are attributed to anti-inflammatory properties of these compounds, making these compounds promising agents as potential new treatment options against *G. vaginalis*-associated vaginal infections.

## 1. Introduction

Bacterial vaginosis (BV) is the predominant vaginal infection in women aged 14 to 49 [1]. It affects approximately 23–29% of women worldwide, with annual expenditures on symptomatic BV treatment reaching $4.8 billion [2]. Bacterial vaginosis is characterized by a substantial reduction in *Lactobacillus* populations, accompanied by a 100–1000 fold increase in the proliferation of facultative or obligate anaerobic microorganisms. These include *Gardnerella vaginalis*, *Megasphaera* spp., *Atopobium vaginae*, *Dialister* spp., *Mobiluncus* spp., *Sneathia amnii*, *S. sanguinegens*, *Porphyromonas* spp., *Prevotella* spp., and various bacteria within the *Clostridiales* order [3,4].

Bacterial vaginosis is often asymptomatic; nevertheless, women with BV are more prone than those without to experience vaginal odor, pruritus, and discharge [5]. Serious adverse health effects linked to bacterial vaginosis encompass heightened infertility risk, negative pregnancy outcomes, the occurrence of sexually transmitted infections (STIs)—such as chlamydia, gonorrhea, infections with human papillomavirus (HPV), and human immunodeficiency virus (HIV)—as well as pelvic inflammatory disease (PID), including endometritis [6,7,8,9]. While the precise etiology of BV remains debated, it is widely believed that most infections are initiated by *Gardnerella vaginalis*, which forms a biofilm that facilitates the proliferation of other opportunistic bacteria [10,11]. *Gardnerella* spp. and other anaerobes that displace commensal lactobacilli form biofilms on the vaginal epithelium during the onset of BV. In contrast to planktonic bacteria, the biofilm of *G. vaginalis* is more resistant to two common agents that are present in healthy vaginal environments: hydrogen peroxide and lactic acid [12]. This leads to an increased pH of vaginal fluid and colonization of the vagina with a heterogeneous polymicrobial community, promoting the progression and further failure of treatment. Currently, there are very few therapeutic options available for bacterial vaginosis, and the majority of cases are managed with antibiotic medication such as oral metronidazole, secnidazole (FDA approved in 2017), vaginal metronidazole gel (FDA approved in 2021), vaginal clindamycin, or tinidazole [1]. However, recurrence rates of BV affect up to 80% of women within nine months after initial treatment [13], and antibiotic resistance is an emerging concern [14]. Experts have advocated adjuvant therapy with probiotics, and several trials have examined the efficacy of probiotics in the treatment of bacterial vaginosis, both as monotherapy and in conjunction with antibiotics [15]. *G. vaginosis* is known to readily develop resistance to metronidazole, forming persistent strains within biofilms or host cells that shield them from antibiotic therapy [16]. Moreover, bacterial resistance and the persistence of infections lead to recurrence and relapse of bacterial vaginosis. To address these challenges, innovative concepts and methodologies distinct from conventional antibiotic mechanisms are necessary for the identification of antibacterial alternatives capable of overcoming bacterial resistance and persistence of infection without inducing new drug resistance or adversely affecting probiotic species [17].

Ceragenins represent a novel class of low molecular-weight antimicrobial agents designed as nonpeptide, cost-effective analogs of endogenous antimicrobial peptides (AMPs). Ceragenins are positively-charged derivatives of cholic acid [18]. Functionally, they mimic endogenous AMPs, which are crucial components of the innate immune system responsible for maintaining mucosal integrity [19]. Previous research has demonstrated the potent antibacterial properties of ceragenins against various pathogens, including resistant organisms, which is based on their ability to disrupt bacterial membranes, eliminate established biofilm and intracellular bacteria, and reduce bacterial adherence [20,21,22,23]. Importantly, our previous studies have shown that the antibacterial spectrum of ceragenins includes human infection-causing anaerobic bacteria, such as *Bacteroides*, *Prevotella*, *Clostridium*, and *Propionibacterium* species [24]. Moreover, the extensive antimicrobial spectrum and antibiofilm properties of these compounds have significant advantages as prospective treatments, particularly in the management of mixed, polymicrobial illnesses, where the efficacy of a broad-spectrum antibiotic is essential. Ceragenins, such as CSA-13, CSA-44, and CSA-131, were selected for their physicochemical properties similar to those of naturally occurring antimicrobial peptides (AMPs) and their broad-spectrum antimicrobial efficacy, confirmed in previous studies. CSA-13 has been thoroughly investigated for its high efficiency against both Gram-positive and Gram-negative bacteria, demonstrating significant effectiveness in biofilm eradication, a critical element in the development of bacterial vaginosis [22,23,24]. Previous studies have demonstrated that CSA-13 exhibits low cytotoxicity and a low tendency for resistance development [25,26], rendering it a suitable option for additional assessment. CSA-44 and CSA-131, analogues of the better-recognized CSA-13, were incorporated in this study to investigate the effects of first- and second-generation ceragenins with respect to their antibacterial activity and safety. These drugs have shown activity against a broad spectrum of microorganisms in previous in vitro experiments; however, their relative effectiveness against *Gardnerella vaginosis* and potential in the treatment of bacterial vaginosis have not been thoroughly investigated. At the same time, the efficacy of ceragenins against *G. vaginalis*, the primary pathogen associated with BV, remains unexplored. Therefore, the purpose of this study was to (i) assess the activity of ceragenins against *G. vaginalis* in vitro and (ii) to evaluate the effectiveness and anti-inflammatory activities of CSA-13 in an *G. vaginalis*-induced BV model. By elucidating the antimicrobial and anti-inflammatory properties of ceragenins, this study aimed to provide insights into their potential as novel therapeutic agents for the management of BV, addressing current treatment limitations and offering a promising alternative for reducing bacterial load and inflammation associated with this common infection.

## 2. Results

### 2.1. Antimicrobial Activity of Ceragenins Against G. vaginalis

MIC and MBC values of three ceragenins (CSA-13, CSA-44, and CSA-131) were determined against *Gardnerella vaginalis* ATCC 49145 and five clinical isolates (S01–S05). The results are summarized in Table 1.

The ceragenins demonstrated strong antimicrobial activity against *G. vaginalis* strains. CSA-131 showed the most potent activity, with MIC values ranging from 0.0625 to 0.25 µg/mL and MBC values from 0.25 to 0.5 µg/mL, consistently outperforming or matching CSA-13 and CSA-44. CSA-13 had MIC values of 0.0625 to 0.25 µg/mL and MBC values from 0.25 to 0.5 µg/mL, while CSA-44 had slightly higher MIC and MBC values. Specifically, CSA-131 achieved the lowest MIC value (0.0625 µg/mL) against the reference strain and clinical isolates, making it the most effective among the ceragenins tested.

### 2.2. Neutral pH May Optimize the Bactericidal Activity of Ceragenin Against G. vaginalis

The results obtained clearly indicate that the antimicrobial activity of ceragenin against *G. vaginalis* depends on the pH (Table 2).

It is noteworthy that at lower pH (4.5 and 5), which is closer to the normal vaginal environment, CSA-13 and CSA-44 showed high antimicrobial activity with MICs of 0.25 µg/mL and an MBC of 0.5 µg/mL. However, when the pH increased to 7—resembling the conditions found in patients with bacterial vaginosis—the efficacy of these ceragenins was greater, with MIC values dropping to 0.125 µg/mL. Importantly, CSA-131 demonstrated the most noticeable pH-dependent variation in its antimicrobial effect. At pH = 4.5 and 5, CSA-131 showed high activity, with an MIC of 0.125 µg/mL and an MBC of 0.25 to 0.5 µg/mL. Importantly, at neutral pH = 7, CSA-131 showed the highest efficacy, with an MIC of 0.0625 µg/mL, suggesting that CSA-131 is particularly potent under alkaline conditions that favor the growth of *G. vaginalis*.

### 2.3. Antimicrobial Efficacy of Ceragenins Against G. vaginalis and Implications for Bacterial Vaginosis Treatment

**Figure 1:**
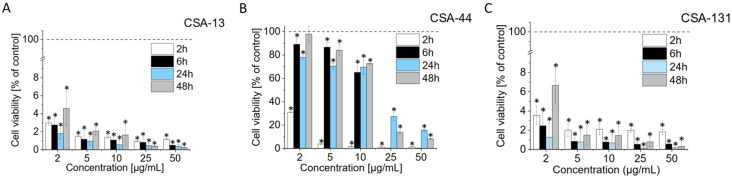
Time-kill activity of ceragenins against *G. vaginalis* ATCC 49145. Viability results were calculated based on luciferase-based assay by collecting bioluminescence signals for CSA-13-, CSA-44-, and CSA-131-treated bacteria (Panels (**A**)–(**C**), respectively). The measurements were made at 2 (white bars), 6 (black bars), 24 (blue bars), and 48 h post-treatment (grey bars). Results are presented as mean ± SD from 3 replicates; * indicates statistical significance ≤ 0.05 when compared to untreated control. The dashed horizontal line indicates untreated control (100% of viability; 0 µg/mL CSA-13, CSA-44, CSA-131).

The findings show that CSA-13 reduces *G. vaginalis* viability in a dose-dependent manner (Figure 1A). At all indicated time points, bacterial viability was significantly decreased, even at low doses of the antimicrobial (2 and 5 µg/mL).

At greater concentrations, bacterial viability clearly decreased as predicted. *G. vaginalis* viability was nearly non-detectable at 25 and 50 µg/mL after 24 and 48 h following CSA-13 treatment, indicating that this compound is useful in lowering the bacterial load over extended exposure periods. Comparable outcomes, with even greater efficacy, were noted with CSA-131 therapy. CSA-131 demonstrated notable efficacy against *G. vaginalis* at all tested concentrations, even low doses (2 and 5 µg/mL)—Figure 1C. After 48 h, bacterial vitality was considerably lower at higher concentrations of 25 µg/mL and 50 µg/mL (bacterial viability varied from 0.79% and 0.32%, respectively). This steady decline of *G. vaginalis* viability at all concentrations and time periods demonstrates the effectiveness of CSA-131 as a powerful antibacterial agent. The effectiveness of CSA-44 against *G. vaginalis* was inconsistent (Figure 1B). Bacterial viability after 48 h remained high at lower doses (2 µg/mL; 98.02%), suggesting limited effectiveness at low concentrations. The effects at 5 µg/mL and 10 µg/mL were comparable. However, after 48 h, a considerable decline in viability was observed at higher concentrations (25 µg/mL and 50 µg/mL), with viability values falling to 13.95% and 8.28%, respectively. This implies that higher doses of CSA-44 are required to achieve a bactericidal effect comparable to that of CSA-13 or CSA-131. Because the continuous suppression of pathogenic bacteria may be required to reestablish a healthy vaginal environment, the capacity of ceragenins, specifically CSA-13 and CSA-131, to retain activity for extended periods of time (up to 48 h) is particularly important in the treatment of bacterial vaginosis.

### 2.4. CSA-13 and CSA-131 Are Highly Effective in Eradicating G. vaginosis Within the Range of Vaginal pH Changes That Might Occur During Treatment of Bacterial Vaginosis

**Figure 2:**
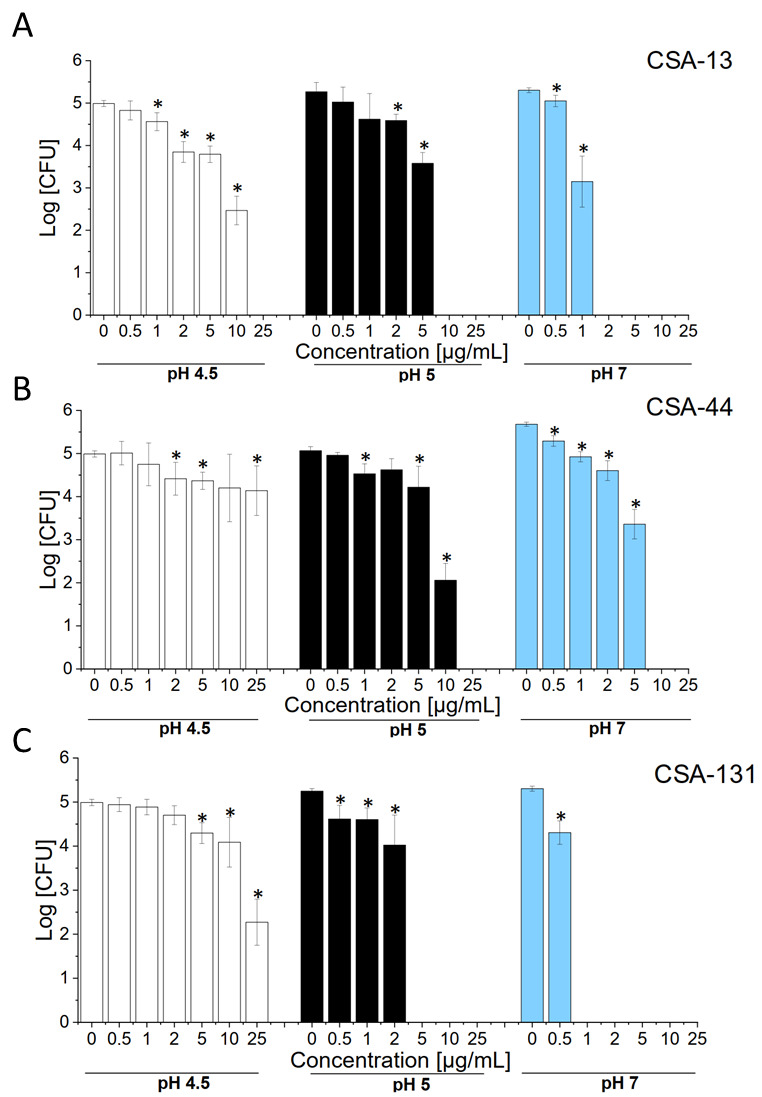
Effect of pH on killing efficiency of CSA-13, CSA-44, and CSA-131 (Panels (**A**)–(**C**), respectively) against *G. vaginalis* ATCC 49145 in planktonic culture. The decline in the survival of *G. vaginalis* culture after incubation with various concentrations of ceragenins at pH 4.5 (white bars), 5 (black bars), and 7 (blue bars) was evaluated using a killing assay method. Results are presented as mean ± SD from 3 replicates; * indicates statistical significance ≤ 0.05 compared to untreated control.

In order to simulate the changing conditions of the vaginal environment during bacterial vaginosis, the effect of ceragenins (CSA-13, CSA-44, and CSA-131) on the viability of *G. vaginalis* ATCC 49145 was evaluated at various pH values (Figure 2). After treatment with different concentrations of ceragenins (0–25 µg/mL), bacterial survival was measured using CFU estimations, allowing for an evaluation of the killing efficacy of tested compounds at pH = 4.5, 5, and 7. *G. vaginalis* viability was dose-dependently reduced by CSA-13, CSA-44, and CSA-131 at pH 4.5, which is similar to the acidic environment of a healthy vagina. At 2 µg/L of CSA-13, the log (CFU) dropped from 4.99 in the untreated control to 3.85. At the same time, a number of live bacteria at 25 µg/mL of CSA-13 dropped to the undetectable limit (Figure 2A).

Similar dose-dependent reductions in bacterial viability were seen at pH = 5, which is more in line with the slightly acidic environment during a bacterial vaginosis episode. At 5 µg/mL of CSA-13, the log (CFU) decreased from 5.27 in the untreated control to 3.58, and no live bacteria were found at concentrations higher than 10 µg/L. Similar effects were observed with CSA-131 treatment (Figure 2C). In agreement with the previous experiments, CSA-44 appeared to be the least effective, as a decrease in *G. vaginalis* viability was not observed until a concentration of 25 µg/mL was administrated (Figure 2B). Importantly, CSA-13 and CSA-131 were considerably more effective at pH 7, which represents a neutral pH that can occur in more severe cases of bacterial vaginosis. For CSA-13, the log (CFU) decreased from 5.30 in the untreated control to 3.15 at 1 µg/L; at 2 µg/L and higher concentrations, total bacterial eradication was obtained (Figure 2A). In contrast, an even better effect was obtained with CSA-131, which completely inhibited the growth of *G. vaginalis* at 1 µg/mL (Figure 2C).

### 2.5. Ceragenins Exhibits Potent, pH-Dependent Anti-Biofilm Activity Against G. vaginalis, with Higher Efficacy Observed at Neutral pH

**Figure 3:**
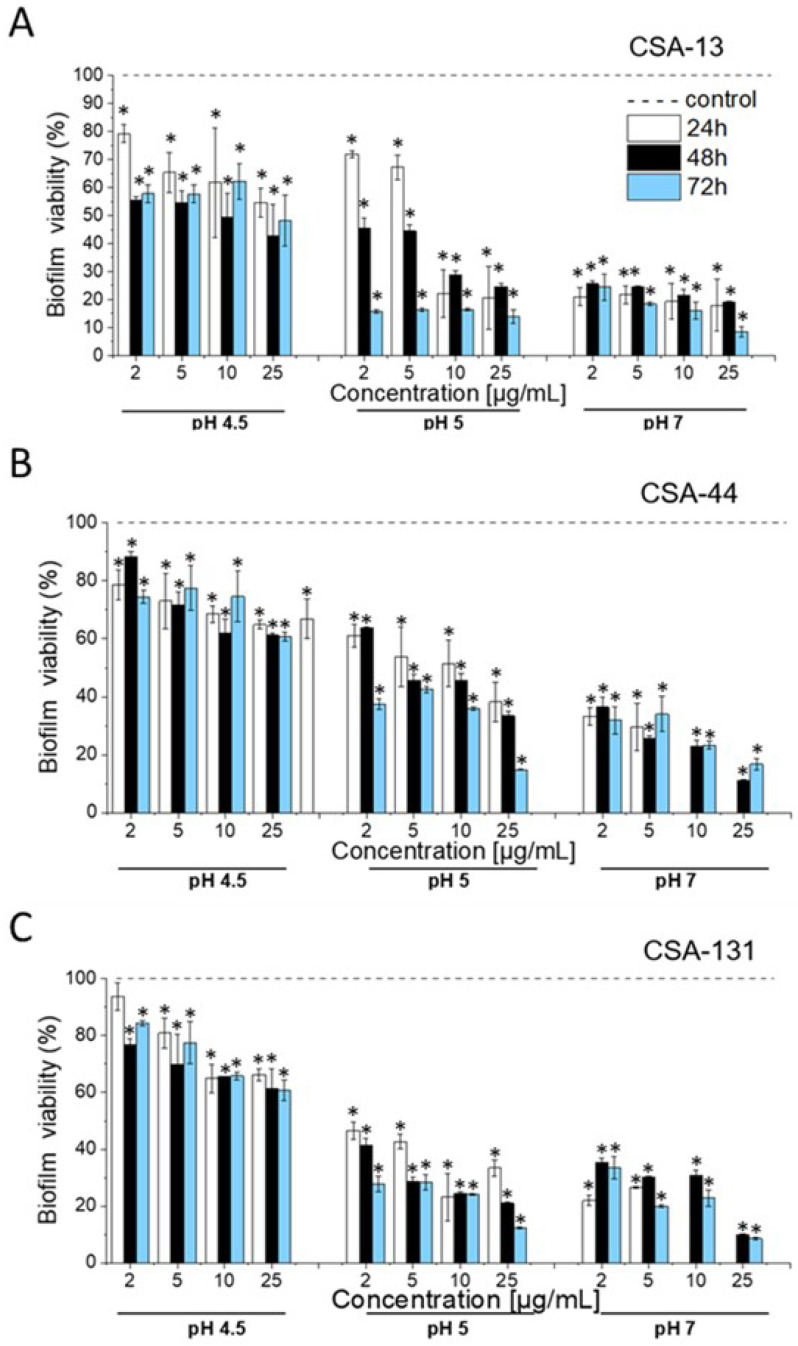
Effect of pH on anti-biofilm properties of CSA-13, CSA-44, CSA-131 (Panels (**A**)–(**C**), respectively) against *G. vaginalis* ATCC 49145. The ability of ceragenins to prevent the biofilm formation of *G. vaginalis* was measured using resazurin at 24 (white bars), 48 (black bars), and 72 h (blue bars). The dashed horizontal line indicates the untreated control (0 µg/mL CSA-13, CSA-44, and CSA-131). Results are presented as mean ± SD from 3 replicates; * indicates statistical significance ≤ 0.05 when compared to untreated control.

Resazurin staining, indicating the viability of bacteria within a biofilm, was used to determine the anti-biofilm effects of ceragenins against *G. vaginalis*. Comparable to planktonic culture-based experiments, at pH 4.5, the tested ceragenins (CSA-13, CSA-44, and CSA-131) showed a moderate, although still significant, reduction in biofilm viability at all time points, with a dose-dependent response (Figure 3).

Upon CSA-13 addition, after 24 h, biofilm viability was reduced from 79.26% at 2 µg/mL to 54.60% at 25 µg/mL (Figure 3A). This reduction persisted for 48 and 72 h, but the decrease in biofilm viability was less pronounced at higher concentrations, suggesting the limited long-term efficacy of not only CSA-13 but also CSA-44 and CSA-131 in highly acidic environments. Notably, at pH = 5, CSA-13 and CSA-131 showed significantly greater activity against biofilm compared to that at pH = 4.5 and also compared to the antibiofilm activity of CSA-44 (Figure 3B). Thus, for example, CSA-13 at a concentration of 2 µg/mL decreased biofilm viability from 71.92% (after 24 h) to 15.8% (after 72 h). Higher concentrations of CSA-13 and CSA-131 resulted in a significant reduction in biofilm viability. When analyzing the results for CSA-13, the viability decreased to 20.5% at 24 h and further decreased to 13.9% at 72 h when a dose of 25 µg/mL was tested for CSA-13. For all ceragenins, the most pronounced anti-biofilm activity was observed at pH = 7. CSA-131 effectively inhibited biofilm viability at all concentrations tested, with the most significant reduction occurring at 10 µg/mL after 24 h, as no detected viability of biofilm-embedded bacteria was observed (Figure 3C). Moreover, CSA-13 at a concentration of 25 µg/mL reduced biofilm viability to 18% after 24 h and to 8.5% after 72 h. These results indicate that ceragenins are most effective at neutral pH, where they can significantly impair biofilm viability for extended periods of time.

### 2.6. At Bactericidal Concentrations, Ceragenins Are Tolerated by HeLa Cells, Suggesting Potential Therapeutic Applications Against G. vaginalis-Induced Bacterial Vaginosis with Minimal Cytotoxic Effects Against Host Cells

**Figure 4:**
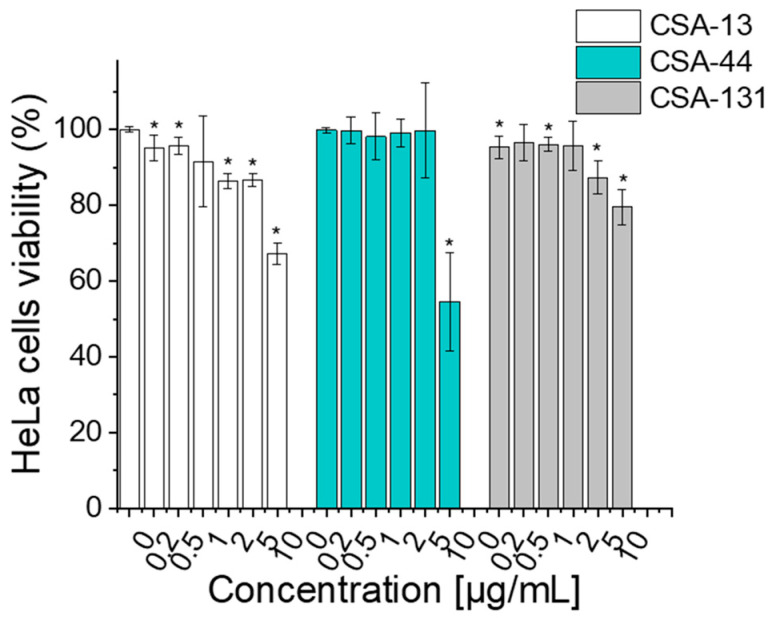
Survival of human cervical cells (HeLa) after incubation with CSA-13, CSA-44, and CSA-131 at doses of 0.2, 0.5, 1, 2, 5, and 10 µg/mL for 24 h. Results are presented as mean ± SD from 3 replicates; * indicates statistical significance ≤ 0.05 when compared to untreated control.

The viability of human cervical cancer cells (HeLa) was assessed following 24 h incubation with ceragenins CSA-13, CSA-44, and CSA-131 at concentrations ranging from 0.2 to 10 µg/mL (Figure 4).

The results showed that CSA-13 did not interfere with cell viability, ranging from 95.1% at a CSA-13 concentration of 0.2 µg/mL to 67.3% at a CSA-13 concentration of 10 µg/mL. Similarly, CSA-44 exhibited a consistent viability profile, remaining above 99% at concentrations up to 5 µg/mL, but showing a significant decline to 54.5% at 10 µg/mL. In contrast, CSA-131 showed a modest decrease in viability, with percentages ranging from 95.3% at 0.2 µg/mL to 79.4% at 10 µg/mL.

### 2.7. High Impact of Ceragenin Treatment in Animal Model of Bacterial Vaginosis

**Figure 5:**
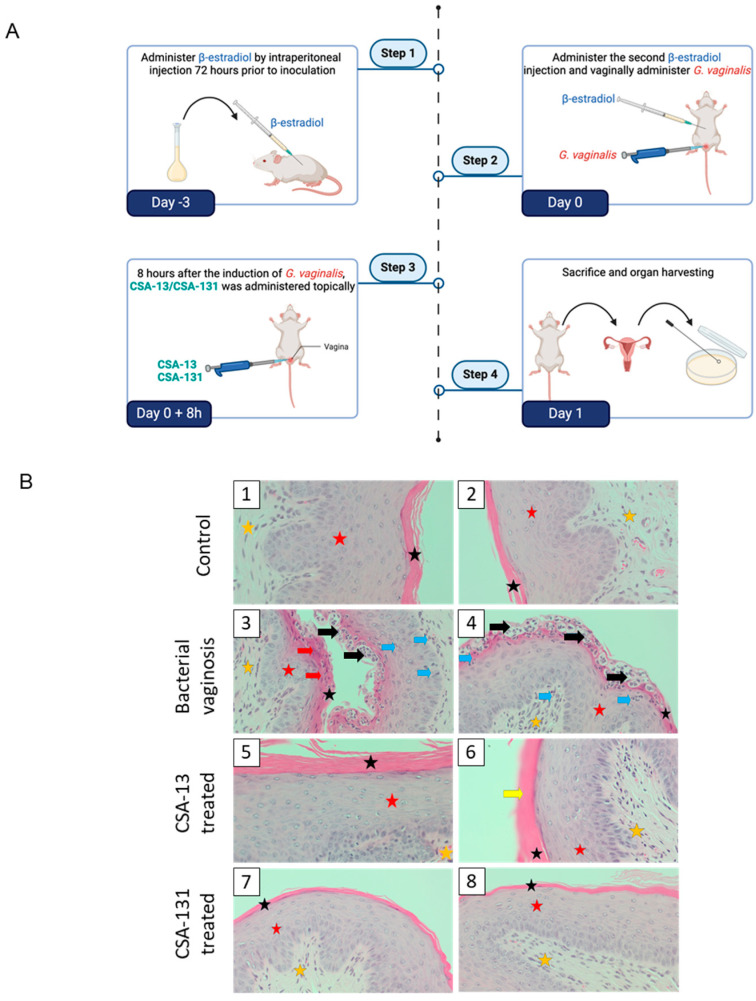
Experimental timeline schematic for the animal experiment of bacterial vaginosis. All mice were intraperitoneally injected with β-estradiol-3-benzoate (1 mg/kg) for 3 days, after which *G. vaginalis* was intravaginally inoculated. After 8 h, mice were treated with CSA-13, CSA-131, or NaCl (control group) (**A**). Representative histopathological images from two different mice in each experimental group, captured at 40× magnification, are shown in hematoxylin- and eosin- (H and E) stained vaginal tissue sections (**B**). The stratum corneum of vaginal epithelium (black star), stratum spinosum of vaginal epithelium (red star), lamina propria of vaginal mucosa (orange star), microabscesses (black arrow), neutrophil infiltration (light blue arrow), parakeratosis and degeneration/acidophilia (red arrow), and hyperkeratosis (yellow arrow) are indicated. Control (1,2), bacterial vaginosis (3,4), bacterial vaginosis and CSA-13 treated (5,6), and bacterial vaginosis and CSA-131 treated (7,8).

To confirm the beneficial effects of ceragenins against *G. vaginalis*, a mouse model of bacterial vaginosis was developed according the protocol summarized on Figure 5A.

Local administration of ceragenins results in a considerable decrease in the bacterial burden and limitation of bacterial vaginosis-associated pathological changes. Histological examination of hematoxylin- and eosin- (H and E) stained vaginal tissue sections provided insights into the morphological changes associated with bacterial vaginosis and the effects of ceragenin treatment (Figure 5B). In the untreated control vaginosis group, there was a marked presence of neutrophil infiltrates within the mucosal stroma, along with pronounced cervical epithelial hyperplasia, indicative of an inflammatory response. In contrast, tissues from all treatment groups exhibited minimal hyperplasia of the cervical epithelium, suggesting that the ceragenins may mitigate hyperplastic changes typically associated with bacterial vaginosis. Specifically, the group treated with CSA-13 demonstrated a reduction in the density of neutrophils within the cervical mucosa compared to the group treated with CSA-131, highlighting a differential effect on inflammation. Notably, the neutrophil infiltration observed in the vaginal mucosa was characterized by features of hyperkeratinization and parakeratosis, which align with the histopathological criteria for bacterial vaginosis. Additionally, while neutrophil infiltration was present in both treated groups, it was evident that both compounds improved the histological appearance of the vaginal tissues. CSA-131 was particularly effective, exhibiting a greater reduction in neutrophil infiltration within the vaginal mucosal epithelium than CSA-13. It is important to note that the observed neutrophil infiltration is likely influenced by the hormonal and ovarian cycles of the female subjects, contributing to the physiological changes in the mucosa of the female reproductive tract.

**Figure 6:**
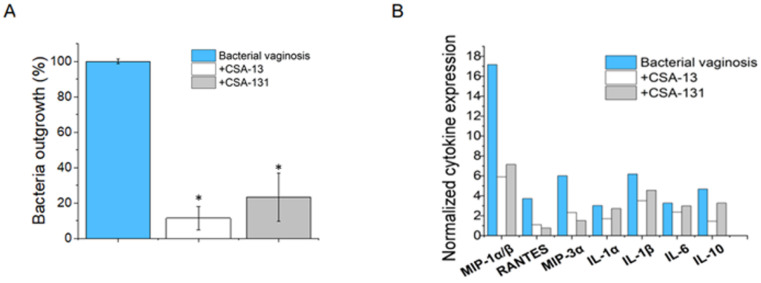
Outgrowth of *G. vaginalis* from homogenates of vaginal tissues collected from mice treated with CSA-13 (white) and CSA-131 (grey) compared to non-treated (blue) mice (**A**). Alterations in inflammation-associated cytokines in the serum of sacrificed animals following the administration of CSA-13 (white) or CSA-131 (grey) compared to untreated infected animals (blue) (**B**). Results are presented as mean ± SD from three replicates; * indicates statistical significance ≤ 0.05 when compared to untreated control.

Ceragenins CSA-13 and CSA-131 significantly reduced the outgrowth of *G. vaginalis* from vaginal tissue homogenates in a mouse model of bacterial vaginosis. As demonstrated in Figure 6A, bacterial outgrowth was significantly reduced to 11.5% following CSA-13 treatment (compared to untreated control animals), showing a strong antimicrobial effect. Treatment with CSA-131 similarly resulted in a reduction, but to a lower amount, with bacterial expansion measuring 23.3%.

These findings indicate that both CSA-13 and CSA-131 have significant antibacterial activity against *G. vaginalis* in vivo, with CSA-13 showing greater efficacy in this setting. The considerable reduction in bacterial load demonstrates the compounds’ potential as treatment agents for bacterial vaginosis.

### 2.8. CSA-13 and CSA-131 Effectively Reduce the Inflammatory Response Associated with Gardnerella vaginalis Infection, as Indicated by the Downregulation of Multiple Cytokines

As demonstrated in Figure 6B, the treatment of infected animals with ceragenins significantly improved the inflammatory profile of the treated animals, suggesting the beneficial effects of these molecules against bacterial vaginosis-associated inflammation. More precisely, CSA-13 significantly reduced the expression of macrophage inflammatory protein (MIP)-1α/β, and RANTES belongs to the β or CC family of chemokines. The results are as follows: MIP)-1α/β (7.1 for CSA-13 treated, 5.9 for CSA-131 treated normalized value vs. 17.2 in *G. vaginalis*-infected group), RANTES (0.8 for CSA-13 treated and 1.1 for CSA-131 treated vs. 3.7 in *G. vaginalis*-infected group), and MIP-3α, with values of 1.5 for CSA-13 treated, and 2.3 for CSA-131 treated vs. 6.0 for *G. vaginalis*-infected group. The pro-inflammatory cytokines IL-1α and IL-1β displayed a distinct trend. CSA-13 therapy reduced IL-1α expression by 2.7 compared to 3.0 in the *G. vaginalis*-infected group, while CSA-131 treatment had a less significant effect (1.7). Both treatments, CSA-13 and CSA-131, lowered IL-1β levels to normalized values of 4.6 and 3.5, respectively, compared to 6.2 in the untreated group. Moreover, serum levels of IL-10 tended to be higher in mice inoculated with *G. vaginalis* compared to the ceragenins treatment groups. The reduction in pro-inflammatory cytokines following treatment with these compounds indicates their potential therapeutic role in mitigating the inflammation associated with bacterial vaginosis.

## 3. Discussion

Our study is a first report describing the efficacy of ceragenins as potential therapeutics against bacterial vaginosis, a condition marked by a transition in vaginal pH from acidic to neutral, which promotes the proliferation of pathogens like *G. vaginalis*. Many BV-associated bacteria are resistant to the current first-line antibiotics, such as clindamycin and metronidazol [27]. Recent studies indicate that the etiological agents in bacterial vaginosis are not only anaerobic bacteria but also aerobic bacteria such as *Streptococcus anginosus*. This makes the treatment of bacterial vaginosis even more challenging, as the first-line antibiotics currently used are effective mostly against anaerobic bacteria [28,29]. This is opposed to ceragenins, which have a strong effect against aerobic and anaerobic bacteria [23,24]. In addition, while metronidazole and clindamycin are often ineffective against biofilm-associated bacteria that persist on the vaginal epithelium and contribute to high recurrence rates [16], ceragenins are promising against both planktonic *G. vaginalis* and its biofilm. Consequently, ceragenins not only provide more general antibacterial coverage but also target resistant biofilms that conventional antibiotics cannot eradicate, thus helping to prevent recurrence.

Ceragenins, which are synthetic antimicrobial agents, exhibit promising pH-dependent efficacy against *G. vaginalis* (both planktonic and biofilms), particularly at neutral pH, making their action most optimal in altered bacterial vaginosis environments. This pH-dependent mechanism resembles that of other cationic peptides, such as LL-37 and human β-defensin-3, which exhibit less efficacy in acidic environments [30]. Researchers’ investigations into the potential of a more alkaline pH to enhance peptide activity have demonstrated that the peptide initially occupies a position parallel to the lipid bilayer, which weakens the membrane prior to its insertion [31,32]. Subsequently, the peptide inserts and disrupts the membrane. The peptide enters the membrane more swiftly due to its lower positive charge at an alkaline pH. Another point worth making is also that at lower pH concentrations, the bacterial membrane may be more stable or less fluid, which may make it more difficult for membrane-targeting antimicrobials to penetrate and disrupt [33]. In a similar manner, cationic peptides and their synthetic counterparts, ceragenins, can interact with the bacterial membrane, resulting in the corresponding effects of alkaline pH. The above may lead to a more efficient disruption of the bacterial membrane, resulting in more effective killing. Given the lower pH, the protonation of bacterial surface molecules, or of the ceragenins themselves, may reduce the response of these interactions, resulting in a less effective disruption of the bacterial membrane, and thus lytic activity. It is worth noting that this does not significantly affect the MIC values, however, as the concentration needed to inhibit growth (not necessarily kill the bacteria) remains in a similar range (Table 2). This reduction in activity of antimicrobial peptides as well as ceragenins at lower pH levels is particularly relevant in the vaginal environment, where maintaining acidity is essential for the growth of beneficial *Lactobacillus* species and thus, maintaining a proper microbiota of the reproductive tract. Given these findings, there is significant potential for future studies to explore combining ceragenins in co-therapy with probiotics for the purpose of improved *Lactobacillus* growth. This approach could help restore and maintain an acidic vaginal environment after the effective eradication of pathogenic bacteria responsible for bacterial vaginosis.

In the present investigation, our method of treating bacterial vaginosis entails the initial elimination of the pathogenic bacteria, followed by the establishment of a healthy vaginal environment. It was found previously that in intestinal disorders, particularly those involving *Clostridium* species, CSA-13 effectively eliminates pathogenic *Clostridium* while concurrently promoting the proliferation of beneficial probiotic bacteria [34]. This optimistic mechanism by which the vaginal microbiome can be similarly restored after the eradication of bacterial vaginosis pathogens is suggested by the dual action of CSA-13, which reduces the pathogenic load and promotes the growth of probiotics microbes. In effect, addressing both the acute infection and the long-term maintenance of vaginal health, this approach has the potential to represent a significant advancement in the management of bacterial vaginosis. Moreover, ceragenins, due to their unique, non-specific, membrane-dependent mechanism of action, are effective against both drug-sensitive and drug-resistant strains, including *Gardnerella vaginalis*. This mechanism allows them to disrupt bacterial membranes in a way that is less likely to promote resistance compared to conventional antibiotics. Thus, ceragenins hold significant potential for reducing recurrence rates and improving treatment outcomes, especially in light of the increasing resistance to antibiotics like metronidazole, where resistance rates for *G. vaginalis* have reached up to 50%.

Our findings indicate that ceragenins exhibit satisfactory biocompatibility at bactericidal concentrations up to 5–10 µg/mL. Although a decrease in the viability of cells might be observed in the higher concentrations of ceragenins (particularly CSA-13 and CSA-44), at doses that are recognized as effective, the viability of cells is approximately 80% or higher. The high cell viability observed after ceragenin treatment highlights their potential as antibacterial agents that spare the host’s epithelial cells—a critical factor given that traditional treatments can damage the vaginal lining, increasing the risk of side effects and recurrent infections. Furthermore, the ability to use different ceragenins based on the severity of the infection and dosing requirements could offer a significant advantage in treatment strategies. For instance, the high viability of HeLa cells after CSA-44 treatment at low doses (0–5 µg/mL) suggests it may be particularly suitable for long-term or preventive use. In contrast, CSA-13 and CSA-131, despite being slightly more cytotoxic at higher concentrations, might be reserved for more severe infections where a stronger antimicrobial effect is needed.

Testing ceragenins using an in vivo model of bacterial vaginosis is an important prelude to assessing the potential use of ceragenin in the treatment of *G. vaginalis* infections. However, it should be kept in mind that bacterial vaginosis in women is a polymicrobial syndrome. In addition, a limitation of the mouse model, which does not fully reflect all aspects of human vaginal physiology, is that the mouse microbiome is not dominated by *Lactobacillus* (which causes reduced lactic acid levels and increased pH). Despite the lack of comparison of the effectiveness of ceragenins with conventional antibiotics in this study, similar comparisons have been published in previous studies that demonstrate the greater effectiveness of ceragenins compared to conventional antibiotics in the treatment of various bacterial infections [23,25,35]. These studies have shown that ceragenins can offer advantages, particularly in terms of their broad-spectrum activity, lower propensity for resistance development, and ability to disrupt biofilms more effectively than traditional antibiotics. In our mouse model of bacterial vaginosis, the histological, microbiological, and inflammation cytokine findings demonstrate the efficacy of these compounds to reduce the bacterial load and the inflammatory response.

The histopathological analysis of animal tissues was conducted by independent pathomorphologists who were blinded to the treatment groups. Histological analysis of haematoxylin- and eosin- (H and E) stained sections of vaginal tissue showed marked inflammatory changes in the untreated control group, characterized by significant infiltration of neutrophils and marked hyperplasia of the cervical epithelium. These features are typical of the inflammatory response associated with bacterial vaginosis, indicating severe mucosal damage and immune activation in the presence of *G. vaginalis* [36]. Neutrophils, being the major cells recruited to the site of infection, play a pivotal role in mediating the inflammatory response against pathogens [37]. Normally present in varying amounts throughout the female reproductive tract, neutrophils increase significantly in response to infection, driven by chemotactic cytokines such as IL-8 [38,39]. These neutrophils penetrate from the vaginal epithelium into the lumen to phagocytize pathogens and cellular debris. Furthermore, they also respond to pathogens by producing oxidative compounds, releasing antimicrobial peptides (AMPs), and cytokines, which further stimulate immune responses and recruit additional immune cells [40]. The presence of hyperkeratinization and parakeratosis further confirms the diagnosis of bacterial vaginosis, highlighting the pathological impact of the infection on the vaginal epithelium. Conversely, the tissues from the groups treated with ceragenin showed a significant decrease in these abnormal characteristics. Both CSA-13 and CSA-131 successfully decreased cervical epithelial hyperplasia and neutrophil infiltration, indicating that these chemicals have a protective effect against the hyperplastic and inflammatory alterations caused by *G. vaginalis*. Significantly, CSA-131 demonstrated a notable ability to decrease the presence of neutrophils in the vaginal mucosal epithelium, suggesting a possibly greater anti-inflammatory characteristic when compared to CSA-13. The differential effect on neutrophil infiltration emphasizes the potential of ceragenins to not only manage infection but also regulate the host immune response, thus minimizing tissue damage and facilitating mucosal repair. It is important to recognize that the presence of neutrophil infiltration in both the treated and untreated groups may be impacted by hormonal and ovarian cycles, which are known to influence the mucosal environment of the reproductive tract [41]. This physiological variability emphasizes the necessity for additional research to comprehensively comprehend the effects of ceragenins during various phases of the hormone cycle of mice.

Notwithstanding the graver repercussions associated with *G. vaginalis* and other bacteria linked to bacterial vaginosis, there is less evidence of an inflammatory response in the vaginal tracts of affected women. As was already mentioned, BV’s designation as an inflammatory or non-inflammatory disorder causes considerable debate [42]. Most instances of BV lack clinical symptoms of overt inflammation, such as swelling and redness [43], which seems at odds with studies that reveal elevated levels of inflammatory cytokines [43,44,45,46,47,48]. Our in vivo experiments demonstrate that *G. vaginalis* may induce significant inflammatory responses within 24 h, characterized by the release of proinflammatory cytokines IL-1α and IL-1 β at levels similar to those seen in bacterial vaginosis [48,49]. IL-1β is synthesized by multiple immune cells in reaction to infection and injury to confer resistance against pathogens [50]. Consequently, our findings demonstrate that *G. vaginalis* elicited an inflammatory response in the vaginal tissues of mice. This observation aligns with prior studies that have documented increased levels of IL-1β in murine models of BV, as well as in patients with BV [51,52,53]. Furthermore, we observed that BV-positive mice had markedly elevated levels of the inflammatory cytokine MIP-1β, which is consistent with studies among women with bacterial vaginosis [54]. In contrast, after treatment with ceragenins, their levels decreased (11-fold and 10-fold for CSA-13 and CSA-131, respectively). Numerous investigations have indicated elevated concentrations of pro-inflammatory cytokines, including IL-1β, IL-6, and IL-8, in vaginal tissues from women with bacterial vaginosis (BV) relative to “healthy” controls [44,46,55,56]. In our study, we found elevated IL-10 levels in a group of mice with bacterial vaginosis, which is consistent with previous studies [53,57]. The reduction of an anti-inflammatory cytokine IL-10 concentration by CSA-13 and CSA-131 in comparison to the control group with bacterial vaginosis necessitates additional clarification. Accordingly, in the presence of ceragenins, the observed decrease in IL-10 may indicate a transition in the immune response to a more pro-inflammatory state, which could potentially improve the host’s capacity to combat the bacterial infection. Furthermore, the decrease in IL-10 may be indicative of the ceragenins’ ability to modify the local cytokine milieu, which could result in a more efficient clearance of *Gardnerella vaginalis* and associated pathogens. In the revised manuscript, we offer a more comprehensive examination of these potential mechanisms and their implications. Conversely, several investigations have not demonstrated links between particular cytokines, including IL-6, and BV [58,59]. In addition to cytokines, the researchers investigated proof of *G. vaginalis*-induced inflammation in the vaginal tissues of infected mice by measuring myeloperoxidase, iNOS, and COX-2 levels [60]. Higher amounts of these inflammatory markers were found in mice injected with *G. vaginalis* than in uninfected controls.

## 4. Materials and Methods

### 4.1. Bacterial Isolates and Antimicrobial Compounds

The study was performed using a laboratory strain of *G. vaginalis* (American Type Culture Collection; ATCC 49145) and five clinical strains of *G. vaginalis* isolated from patients with confirmed bacterial vaginosis. Clinical vaginal specimens were cultured anaerobically at 37 °C after being plated onto selective *G. vaginalis* agar plates (Thermo Scientific™, Waltham, MA, USA). *G. vaginalis* was identified as small, transparent colonies that showed Gram-variable, pleomorphic, coccobacillary morphology upon Gram staining. Ceragenins (CSA-13, CSA-44 and CSA-131) were synthesized as previously described [61].

### 4.2. Determination of Minimal Inhibitory and Bactericidal Concentrations of Ceragenins Against Gardnerella vaginalis Using Microdilution Method

Using the microdilution method, the minimal inhibitory (MIC) and minimal bactericidal (MBC) concentrations of tested ceragenins (CSA-13, CSA-44, CSA-131) against *G. vaginalis* ATCC 49145 and five clinical strains of *G. vaginalis* (labeled as S01, S02, S03, S04, S05) were determined in BHI (Brain Heart Infusion) broth at concentrations ranging from 0.0625 to 16 µg/mL. Incubation was performed anaerobically at 37 °C. Aliquots from each overnight MIC dilution series were quantitatively plated on selective *G. vaginalis* agar plates for MBC measurement. The lowest concentration of ceragenins (MBC) reduced viable counts by at least 99.9%.

### 4.3. Effect of pH on the Antimicrobial Activity of Ceragenins

In the first step, the procedure described above was employed to estimate MICs and MBCs of ceragenins against *G. vaginalis* ATCC 49145 in the same conditions with varied pH (pH = 4.5, 5 and 7). In the second step, a colony counting assay (killing assay) was performed to evaluate the bactericidal activity of the ceragenins (CSA-13, CSA-44, and CSA-131) against a laboratory strain of *Gardnerella vaginalis*, ATCC 49145, in PBS at pH = 4.5, 5, and 7. Briefly, individual colonies of *G. vaginalis* were resuspended in sterile deionized water to a concentration of approximately 10^8^ colony-forming units per milliliter (CFU/mL). The bacterial suspension was then diluted to a final concentration of 10^5^ CFU/mL. The killing assay was conducted by exposing the bacterial cultures to various concentrations of the ceragenins, ranging from 0 to 25 μg/mL following an incubation in an anaerobic atmosphere (60 min at 37 °C). The evaluation was brought to an end by placing the plates on ice. Samples were then serially diluted from 10-fold to 1000-fold. A 10 μL aliquot from each dilution was plated onto selective agar for *G. vaginalis*. The agar plates were cultured anaerobically at 37 °C. The number of colonies was counted to determine the colony-forming units per milliliter (CFU/mL) of each sample. In the third step, the anti-biofilm activity of ceragenins (CSA-13, CSA-44, and CSA-131) against biofilm-embedded *G. vaginalis* ATCC 49145 was evaluated using resazurin-based fluorometric staining. For this assay, *G. vaginalis* at the logarithmic phase of growth was suspended in BHI broth with 1% glucose to an optical density (OD) of approximately 0.1. The bacterial suspension was then diluted 50-fold in BHI supplemented with 1% glucose and adjusted to pH = 4.5, 5, and 7, and then distributed into 96-well flat-bottom plates to allow biofilm to form. Biofilms were exposed to the tested ceragenins at concentrations ranging from 0 to 25 µg/mL for 24, 48, and 72 h and anaerobically grown at 37 °C. After incubation, planktonic (free-growing) cells were carefully removed, leaving only the biofilm-embedded bacteria. Resazurin, a viability indicator, was then added to each well at a final concentration of 200 µg/mL. The biofilm viability was assessed by measuring the fluorescence intensity at excitation/emission wavelengths of 520/590 nm using a Varioskan Lux microplate reader upon 1 h incubation (ThermoFisher Scientific, Waltham, MA, USA).

### 4.4. Time-Kill Assay

The BacTiter-Glo™ Luminescent Cell Viability Assay (Promega, Madison, WI, USA) was used to determine the time-kill activity of the tested ceragenins (CSA-13, CSA-44, and CSA-131) in the concentration range of 0–50 µg/mL against *Gardnerella vaginalis* ATCC 49145. Briefly, 96-well clear-bottom black plates were filled with 200 μL of each serial dilution, and bacteria were exposed to the ceragenins for 2, 6, 24, and 48 h while incubating anaerobically at 37 °C. After incubation, the plates were centrifuged, and 50 μL of BacTiter-Glo reagent was added to the medium. The bioluminescence signal was then analyzed to assess bacterial viability. The number of live bacteria was directly correlated with the bioluminescence output. Luminescence was measured using a Varioskan™ LUX multimode microplate reader (Thermo Fisher Scientific, Waltham, MA, USA).

### 4.5. Cell Culture

Human cervical cancer cells HeLa (CCL-2™, ATCC) were maintained in Dulbecco’s Modified Eagle Medium (DMEM) supplemented with 10% fetal bovine serum (FBS), 50 U/mL penicillin, and 50 μg/mL streptomycin at 37 °C in a 5% CO_2_ incubator.

### 4.6. Assessment of Ceragenins Toxicity Toward HeLa Cells

HeLa cells were seeded in a 48-well plate at a density of 15–20 × 10^3^ cells per well and treated with different concentrations of ceragenins (range 0–10 µg/mL) for a period of 24 h. After ceragenin (CSA-13, CSA-44, CSA-131) treatment, the cells were washed with phosphate-buffered saline (PBS) and incubated with a methylthiazoletetrazole (MTT) solution at a final concentration of 0.5 mg/mL for 4 h. Upon solubilization of formazan precipitate with DMSO, the spectrophotometric absorbance was measured at 550 nm using a Varioskan Lux microplate reader, with untreated cells considered as 100% viable.

### 4.7. Animal Model of Bacterial Vaginosis

The study was carried out in accordance with a previously published method, with some modifications [62]. Female C57/Bl6 mice (6–8 weeks old) were administered with 100 µL of filter-sterilized sesame oil mixed with 0.5 mg of β-estradiol intraperitoneally three days before and on the day of the bacterial injection. Administration of β-estradiol allowed suppression of the inflammatory response and increased susceptibility to *G. vaginalis* colonization in the mouse vagina. The mice were anesthetized using isoflurane and then vaginally injected with approximately 5 × 10^7^ CFU of *G. vaginalis* ATCC 49145 in 20 µL of sterile PBS (OD_600_ = 5.0). Eight hours after the administration of *G. vaginalis*, the mice were treated vaginally with ceragenins (CSA-13 and CSA-131) by administering 100 µL of the ceragenins at a concentration of 20 µg/mL. Twenty-four hours after the treatment, the mice were sacrificed to collect their uterine horns and vaginas. Vaginal samples were collected from each of the following groups: (i) healthy control (n = 5), (ii) bacterial vaginosis induced (n = 5), (iii) bacterial vaginosis treated with CSA-13 (n = 5), and (iv) bacterial vaginosis treated with CSA-131 (n = 5). For the purpose of bacterial outgrowth quantification, the collected tissues were freshly homogenized and plated on growth-selective *G. vaginalis* agar plates, which were then incubated for 48 h under anaerobic conditions. In addition, residual vaginal tissue and uterine horns from each mouse were preserved in 10% buffered formalin phosphate at room temperature for histopathological analysis. Hematoxylin and eosin (H&E) staining was performed, and histological slides were prepared and recorded. Blood was also collected via cardiac puncture into a 2 mL Eppendorf tube and centrifuged for 15 min at 4000 rpm to separate serum, which was used to measure serum cytokine concentrations.

### 4.8. Alterations in Inflammation-Associated Cytokine Levels

Blood was collected from each mouse heart after the experiments were completed. After centrifuging the blood for 10 min at 1800× *g*, the serum was stored at −80 °C for cytokine examination. Serum cytokine levels were analyzed using the Mouse XL Cytokine Array (R&D Systems, Minneapolis, MN, USA) according to the manufacturer’s instructions. Serum samples (40 µL per mouse from each group) from the untreated (bacterial vaginosis; n = 5), CSA-13-treated (n = 5), and CSA-131-treated (n = 5) groups were pooled for each test to obtain a final volume of 200 µL for each array membrane. The arrays were analyzed using the LI-COR Odyssey^®^ Infrared Imaging System, and the results were quantified using Image Studio software (ver. 5.2, USA).

### 4.9. Statistical Analysis

Data are presented as mean ± SD. The significance of differences was determined using the two-tailed Student’s *t*-test. Statistical analyses were performed using OriginPro 2021 (OriginLab Corporation, Northampton, MA, USA). *p* < 0.05 was considered to be statistically significant.

## 5. Conclusions

Ceragenins are a prospective agent for the treatment of *G. vaginalis*-associated vaginal infections due to their antibacterial and anti-inflammatory properties, which are further attributed to their beneficial effects. Although the exact molecular mechanisms are not yet fully understood, our findings as well as those of previous studies suggest that ceragenins have great potential to limit the growth of *G. vaginalis* and other pathogens involved in the development of BV. Future research should expand and optimize polyinfection animal models to refine our understanding of ceragenins’ action and explore the potential for co-administration with probiotics.

## Figures and Tables

**Table 1:** Minimal inhibitory and bactericidal concentrations (MIC/MBC; μg/mL) of CSA-13, CSA-44, and CSA-131 against the *G. vaginalis* ATCC 49145 strain and five clinical isolates (S01—strain number 1, S02 strain number 2, S03—strain number 3, S04—strain number 4, and S05—strain number 5).

	CSA-13	CSA-44	CSA-131
*G. vaginalis* ATCC 49145	0.125/0.25	0.125/0.25	0.0625/0.5
*G. vaginalis* S01	0.125/0.25	0.25/0.5	0.125/0.25
*G. vaginalis* S02	0.125/0.25	0.5/1	0.0625/0.5
*G. vaginalis* S03	0.25/0.5	0.5/1	0.25/0.5
*G. vaginalis* S04	0.0625/0.5	0.5/1	0.0625/0.5
*G. vaginalis* S05	0.0625/0.5	0.25/0.5	0.125/0.25

**Table 2:** Minimal inhibitory and bactericidal concentrations (MIC/MBC; μg/mL) of CSA-13, CSA-44, and CSA-131 against *G. vaginalis* ATCC 49145, recorded at pH of 4.5, 5, and 7.

	pH 4.5 MIC/MBC	pH 5 MIC/MBC	pH 7 MIC/MBC
CSA-13	0.25/0.5	0.25/0.5	0.125/0.25
CSA-44	0.25/0.5	0.25/0.5	0.125/0.25
CSA-131	0.125/0.25	0.125/0.5	0.0625/0.5

## Data Availability

The original contributions presented in the study are included in the article; further inquiries can be directed to the corresponding author.
